# Body Mass Index Changes and Femur Fracture Risk in Parkinson's Disease: National Cohort Study

**DOI:** 10.1002/jcsm.13860

**Published:** 2025-06-04

**Authors:** Sung‐Ho Ahn, Hye Sun Lee, Jun‐Hyuk Lee

**Affiliations:** ^1^ Department of Family Medicine Gangnam Severance Hospital, Yonsei University College of Medicine Seoul Republic of Korea; ^2^ Department of Research Affairs, Biostatistics Collaboration Unit Yonsei University College of Medicine Seoul Republic of Korea; ^3^ Department of Family Medicine Nowon Eulji Medical Center, Eulji University School of Medicine Seoul Republic of Korea

**Keywords:** BMI, cohort, femur fracture, Parkinson's disease, trajectory

## Abstract

**Background:**

Parkinson's disease (PD) increases fracture risk owing to postural instability and bone fragility, with femur fractures being the most frequent and clinically significant. Many patients with PD experience weight loss as the disease progresses, and low body mass index (BMI) is a well‐established fracture risk factor. However, the relationship between longitudinal BMI changes and femur fracture risk in PD remains unclear. We investigated this association using nationwide cohort data.

**Methods:**

This retrospective cohort study used data from the Korean National Health Insurance Service (2009–2014). Overall, 19 422 patients newly diagnosed with PD who underwent three consecutive biennial health screenings were included in the analysis. Based on BMI measurements collected over a median exposure period of 4.01 years (2009–2014), changes were identified using Gaussian finite mixture modelling, classifying participants into two groups: stable BMI (*n* = 16 839) and decreasing BMI (*n* = 2583). The primary outcome was new‐onset femur fracture, defined as hospitalization with the International Classification of Diseases (Tenth Revision) code S72, identified between 2015 and 2022. Multivariable Cox proportional hazard regression analysis was used to estimate hazard ratios (HRs) and 95% confidence intervals (CIs) for new‐onset femur fracture in the decreasing BMI group compared to the stable group.

**Results:**

The mean age of participants was 65.6 ± 8.8 years, 57.0% were women, and the average baseline BMI was 24.2 ± 3.0 kg/m^2^. Compared with the stable group, the decreasing BMI group was older, had a higher baseline BMI, and a lower proportion of current drinkers and regular exercisers. The proportion of women and the prevalence of obesity, hypertension, type 2 diabetes, dyslipidaemia and osteoporosis were also higher in the decreasing group. During a median follow‐up of 8.46 years, 1156 femur fractures occurred. The incidence rate was higher in the decreasing BMI group than in the stable group (10.50 vs. 7.58 per 1000 person‐years). In the unadjusted analysis, the decreasing BMI group exhibited a significantly increased risk of femur fractures (HR 1.41, 95% CI: 1.21–1.65, *p* < 0.001). The association remained significant after multivariable adjustment, with an HR of 1.20 (95% CI: 1.02–1.41, *p* = 0.027).

**Conclusions:**

Patients with PD who experience a decline in BMI over time have a higher risk of femur fracture than those with a stable BMI. Monitoring longitudinal changes in BMI among patients with PD may serve as a practical tool for the early identification of fracture risk and the implementation of preventive strategies.

## 1 Introduction

Parkinson's disease (PD) is the second most common neurodegenerative disorder after Alzheimer's disease [1]. Patients with PD are at high risk of falls and fractures owing to postural instability and gait disturbances [2, 3]. Recent large‐scale cohort studies have shown that approximately one‐third of patients with PD experience some form of fracture during follow‐up [4], and some cases have even been reported as early as the prodromal stage [5].

In particular, femur fracture is the most common and most severe type of fracture among patients with PD, who face a two‐fold higher risk compared to those without PD [6, 7]. Additionally, patients with PD have twice the risk of mortality following femur fracture and are associated with increased surgical complications, including dislocation and revision surgery [6]. Consequently, the prevention of fracture has become a critical therapeutic target in PD care. Clinical strategies such as osteoporosis screening and treatment [8], fall‐prevention exercise programmes, environmental modifications [9] and pharmacologic interventions targeting bone metabolism [10] have emerged as essential components of PD management. These approaches reflect a shift beyond traditional treatment focused on motor symptom control with dopaminergic agents towards a more integrated and multidisciplinary approach [11, 12].

Low body mass index (BMI) or underweight has been linked to an elevated risk of fractures owing to factors such as reduced bone mineral density and impaired balance [13, 14, 15]. Many individuals with PD experience involuntary weight loss and muscle deterioration as their disease progresses, suggesting that those who experience gradual weight loss might be at greater fracture risk [16]. Thus, serial monitoring of BMI trajectories could serve as a straightforward and practical clinical approach to identify patients with PD at increased fracture risk, facilitating timely preventive interventions and improving patient outcomes. However, the link between longitudinal BMI changes and fracture risk in patients with PD remains unclear. To address this gap, we investigated the relationship between BMI changes over time and fracture risk in patients with PD, focusing on femur fractures due to their significant clinical consequences.

Using a nationwide cohort from the Korean National Health Insurance Service (NHIS) Database, we identified distinct longitudinal BMI trajectory patterns among patients with PD by applying data‐driven latent trajectory classification based on Gaussian finite mixture models. Subsequently, we analysed the association between these BMI trajectory patterns and subsequent femur fracture risk.

## 2 Methods

### 2.1 Study Design and Population

We analysed data from the 2009–2014 Korean NHIS health screening programme. The NHIS covers approximately 97% of the Korean population, providing nationwide healthcare utilization data, thereby minimizing selection bias and maximizing generalizability [17]. It offers free biennial national health screening to adults aged ≥ 18 years.

Patients newly diagnosed with PD between 2009 and 2014 were identified using the International Classification of Diseases, Tenth Revision (ICD‐10) G20 code, along with the PD‐specific insurance claim code (V124), which is issued only after clinical confirmation of PD by board‐certified neurologists or neurosurgeons. Registration for this programme requires a physician's certification, and the diagnostic definition has been widely adopted in a previous epidemiological study, reflecting its clinical reliability [18]. The 2009–2014 period was defined as the exposure window to assess BMI trajectory patterns, while the subsequent 2015–2022 period served as the accrual window for identifying new‐onset femur fractures.

**FIGURE 1:**
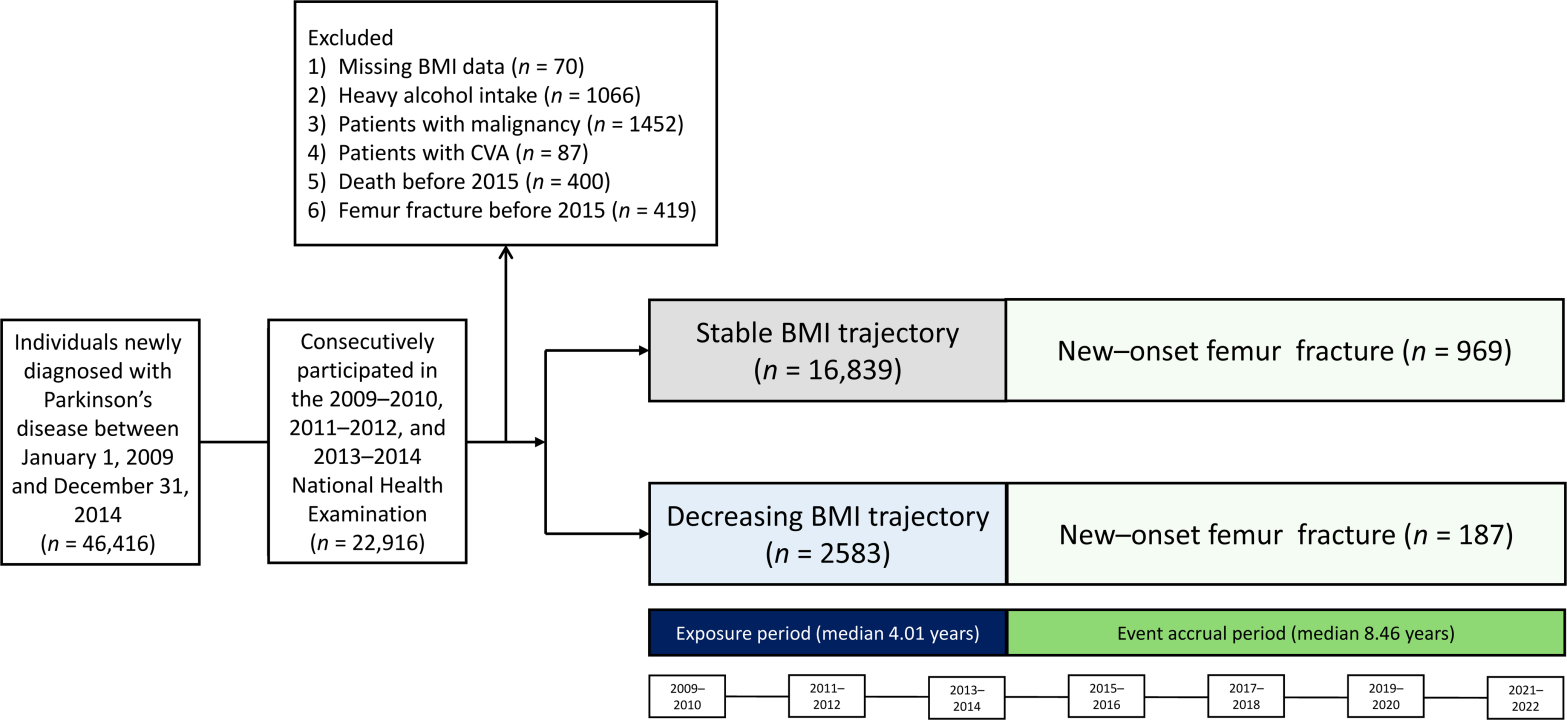
Flowchart of the study population selection. BMI, body mass index.

Figure 1 shows a flowchart of the study population. Among 46 416 individuals newly diagnosed with PD between 2009 and 2014, we selected 22 916 who consecutively participated in the 2009–2010, 2011–2012 and 2013–2014 national health examination. We excluded individuals with missing BMI data (*n* = 70), heavy alcohol intake (*n* = 1066), malignancy before 2015 (*n* = 1452), stroke before 2015 (*n* = 87), death before 2015 (*n* = 400) or femur fracture before 2015 (*n* = 419). The final analytical cohort consisted of 19 422 patients with PD.

This study was approved by the Institutional Review Board of Eulji University Hospital (2023‐09‐009) and was conducted in accordance with the Declaration of Helsinki. Informed consent was waived because we used anonymized data provided by the NHIS database in accordance with the Personal Data Protection Act.

### 2.2 Identification of BMI‐Changing Patterns

**FIGURE 2:**
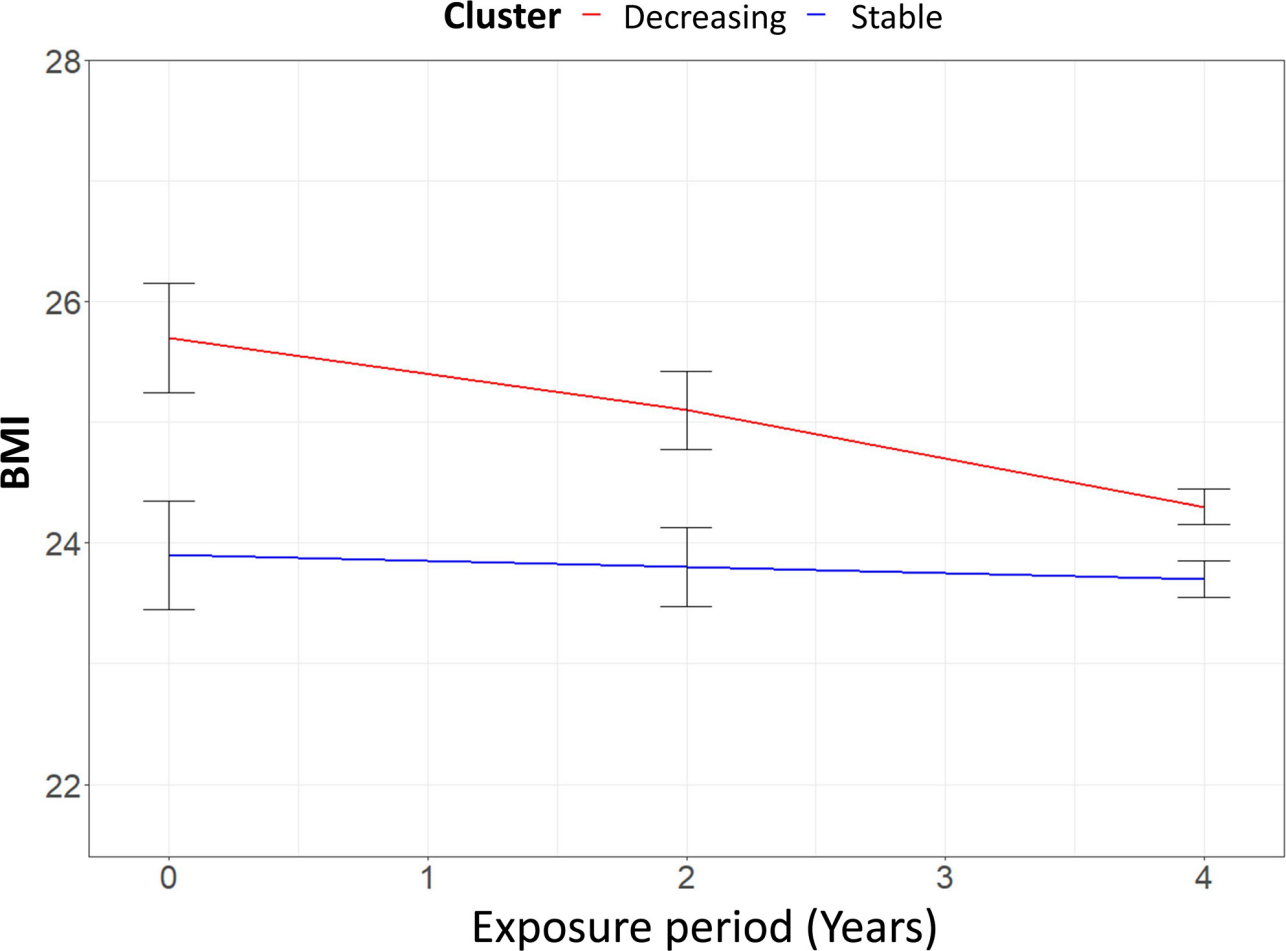
BMI trajectory groups during the exposure period. BMI, body mass index.

During the median 4.01 years of the exposure period, BMI trajectories were identified using Gaussian finite mixture modelling implemented with the R package ‘latrend’ [19]. The optimal trajectory model was selected based on the following criteria: (1) a low Bayesian information criterion, (2) a minimum group size of at least 5% of the total sample and (3) high average posterior probabilities of group membership across trajectory groups, ensuring adequate discriminative ability of the model, indicating strong discriminative performance (Table S1). Based on this approach, participants were classified into two trajectory groups: stable (*n* = 16 839) and decreasing (*n* = 2583) BMI patterns (Figure 2).

### 2.3 Primary Outcome

The primary outcome was a new‐onset femur fracture during the accrual period, defined by the ICD‐10 code S72 and a hospital admission code. The duration for outcome assessment was calculated as the interval between the participant's last health screening (in 2013–2014) and the date of femur fracture, death or the end of follow‐up (31 December 2022), whichever came first. Previous validation studies using the NHIS database demonstrated positive predictive values exceeding 90% for major outcomes such as cardiovascular diseases and cancers [20, 21].

### 2.4 Covariates

Individuals receiving medical aid or in the lowest income quintile, based on NHIS premiums, were classified as low‐income. Smoking status was categorized as current smokers and nonsmokers. Heavy alcohol intake was defined as alcohol consumption of ≥ 30 g/day for men and ≥ 20 g/day for women, respectively. Individuals without heavy alcohol intake were categorized as either current drinkers or nondrinkers. Regular physical activity was defined as vigorous exercise for at least 3 days per week or moderate exercise for at least 5 days per week. Height and weight were measured, and BMI was calculated as weight in kilograms divided by the square of height in meters (kg/m^2^). Obesity was defined, according to the Korean Society for the Study of Obesity, as a BMI of ≥ 25 kg/m^2^ [22]. Blood pressure, including systolic and diastolic blood pressure (SBP and DBP, respectively) readings, was measured after the participants rested in a seated position for 5 min. After a minimum fasting period of 8 h, laboratory examinations included measuring the levels of fasting blood glucose (FBG), total cholesterol, triglycerides, high‐density lipoprotein (HDL) cholesterol and low‐density lipoprotein (LDL) cholesterol. Type 2 diabetes mellitus (T2DM) was defined as an FBG level of ≥ 126 mg/dL or a diagnosis based on ICD‐10 codes E11–E14, with current use of anti‐diabetic medication [23]. Hypertension (HTN) was diagnosed if SBP was ≥ 140 mmHg, DBP was ≥ 90 mmHg or if the individual had ICD‐10 codes I10–I13 or I15, along with current treatment of anti‐hypertensive medications [24]. Dyslipidaemia (DLD) was identified by a total cholesterol level of ≥ 240 mg/dL or by ICD‐10 code E78, with ongoing treatment using lipid‐lowering medications [25]. Osteoporosis was defined by ICD‐10 codes M80–M82 [26]. Stroke was defined as ICD‐10 codes I60–I61 and I63–I64, along with hospital admission [27].

### 2.5 Statistical Analysis

All data are presented as mean ± standard deviation or median (25th and 75th percentiles) for continuous variables and as numbers (percentage, %) for categorical variables. Based on the results of the Kolmogorov–Smirnov test, group differences were assessed using either an independent *t*‐test or Mann–Whitney *U* test for continuous variables and the chi‐squared test for categorical variables.

Kaplan–Meier survival curves were used to depict the cumulative incidence rate of femur fractures between the stable and decreasing BMI groups, with comparisons performed using the log‐rank test. Cox proportional hazards regression analysis was also used to calculate hazard ratios (HRs) and 95% confidence intervals (CIs) for the decreasing BMI group, using the stable group as the reference. In Model 1, demographic variables, including age, sex and BMI at baseline, were adjusted for. Model 2 was additionally adjusted for lifestyle factors such as smoking status, alcohol consumption and physical activity level, in addition to the variables in Model 1. Model 3 was further adjusted for comorbidities, including T2DM, HTN, DLD and osteoporosis, on top of the variables in Model 2. Subgroup analyses were conducted to evaluate the potential effect modification of sex, age (< 65 or ≥ 65 years), obesity, regular exercise and osteoporosis.

All statistical analyses were performed using R (version 4.0.3; R Foundation for Statistical Computing, Vienna, Austria) and SAS Enterprise Guide version 7.1 (SAS Institute, Cary, NC, USA). A two‐sided *p* < 0.05 was considered statistically significant.

## 3 Results

### 3.1 Baseline Characteristics

Table 1 shows the baseline clinical characteristics of the study population by BMI trajectory group. In the total population, the mean age was 65.6 ± 8.8 years, 57.0% were women, and the baseline BMI was 24.2 ± 3.0 kg/m^2^. The decreasing group had a significantly higher baseline BMI than the stable group (25.8 ± 4.1 vs. 24.0 ± 2.7 kg/m^2^; *p* < 0.001). Compared with the stable BMI group, individuals in the decreasing group were older and had a lower proportion of current drinkers and regular exercisers (all *p* < 0.001). They also exhibited a higher median triglyceride level (*p* < 0.001), as well as higher mean values for waist circumference, SBP, DBP, FBG, total cholesterol (all *p* < 0.001) and LDL cholesterol level (*p* = 0.007). In contrast, their HDL cholesterol level was lower compared to the stable group (*p* = 0.024). The proportion of women and the prevalence of obesity, HTN, T2DM, DLD and osteoporosis were higher in the decreasing BMI group (*p* < 0.001 for the proportion of women, the prevalence of obesity, HTN, T2DM and osteoporosis; *p* = 0.001 for DLD).

**TABLE 1:** Baseline characteristics of the study population.

Variables	BMI trajectory groups			
	Stable (*n* = 16 839)	Decreasing (*n* = 2583)	Total (*n* = 19 422)	*p*
Men, *n* (%)	7615 (45.2%)	730 (28.3%)	8345 (43.0%)	< 0.001
Age, year	65.4 ± 8.8	67.5 ± 8.5	65.6 ± 8.8	< 0.001
Obesity, *n* (%)	5947 (35.3%)	1486 (57.5%)	7433 (38.3%)	< 0.001
BMI, kg/m^2^	24.0 ± 2.7	25.8 ± 4.1	24.2 ± 3.0	< 0.001
WC, cm	82.5 ± 8.0	86.4 ± 9.6	83.0 ± 8.3	< 0.001
SBP, mmHg	126.3 ± 15.7	128.7 ± 16.5	126.7 ± 15.8	< 0.001
DBP, mmHg	77.1 ± 9.9	78.3 ± 10.1	77.2 ± 10.0	< 0.001
Current smoker, *n* (%)	1188 (7.1%)	160 (6.2%)	1348 (7.0%)	0.112
Current drinker, *n* (%)	3362 (20.3%)	334 (13.1%)	3696 (19.3%)	< 0.001
Regular exerciser, *n* (%)	3890 (23.3%)	390 (15.2%)	4280 (22.2%)	< 0.001
FBG, mg/dL	102.7 ± 25.4	106.2 ± 32.0	103.1 ± 26.4	< 0.001
Total cholesterol, mg/dL	195.1 ± 38.6	198.6 ± 40.3	195.5 ± 38.8	< 0.001
Triglycerides, mg/dL	114 (82, 162)	124 (89, 173)	116 (83, 163)	< 0.001
HDL cholesterol, mg/dL	52.6 ± 13.4	51.9 ± 13.8	52.5 ± 13.5	0.024
LDL cholesterol, mg/dL	115.8 ± 34.6	117.9 ± 36.8	116.1 ± 34.9	0.007
HTN, *n* (%)	7359 (43.7%)	1383 (53.5%)	8742 (45.0%)	< 0.001
T2DM, *n* (%)	2938 (17.5%)	552 (21.4%)	3490 (18.0%)	< 0.001
DLD, *n* (%)	2598 (15.4%)	467 (18.1%)	3065 (15.8%)	0.001
Osteoporosis, *n* (%)	6705 (39.8%)	1253 (48.5%)	7958 (41.0%)	< 0.001

*Note:* All data are presented as mean ± standard deviation or median (25th and 75th percentiles) for continuous variables and as numbers (percentage, %) for categorical variables.

Abbreviations: BMI, body mass index; DBP, diastolic blood pressure; DLD, dyslipidaemia; FBG, fasting blood glucose; HDL, high‐density lipoprotein; HTN, hypertension; LDL, low‐density lipoprotein; SBP, systolic blood pressure; T2DM, type 2 diabetes mellitus; WC, waist circumference.

### 3.2 Risk of Femur Fracture Based on BMI Changes

**FIGURE 3:**
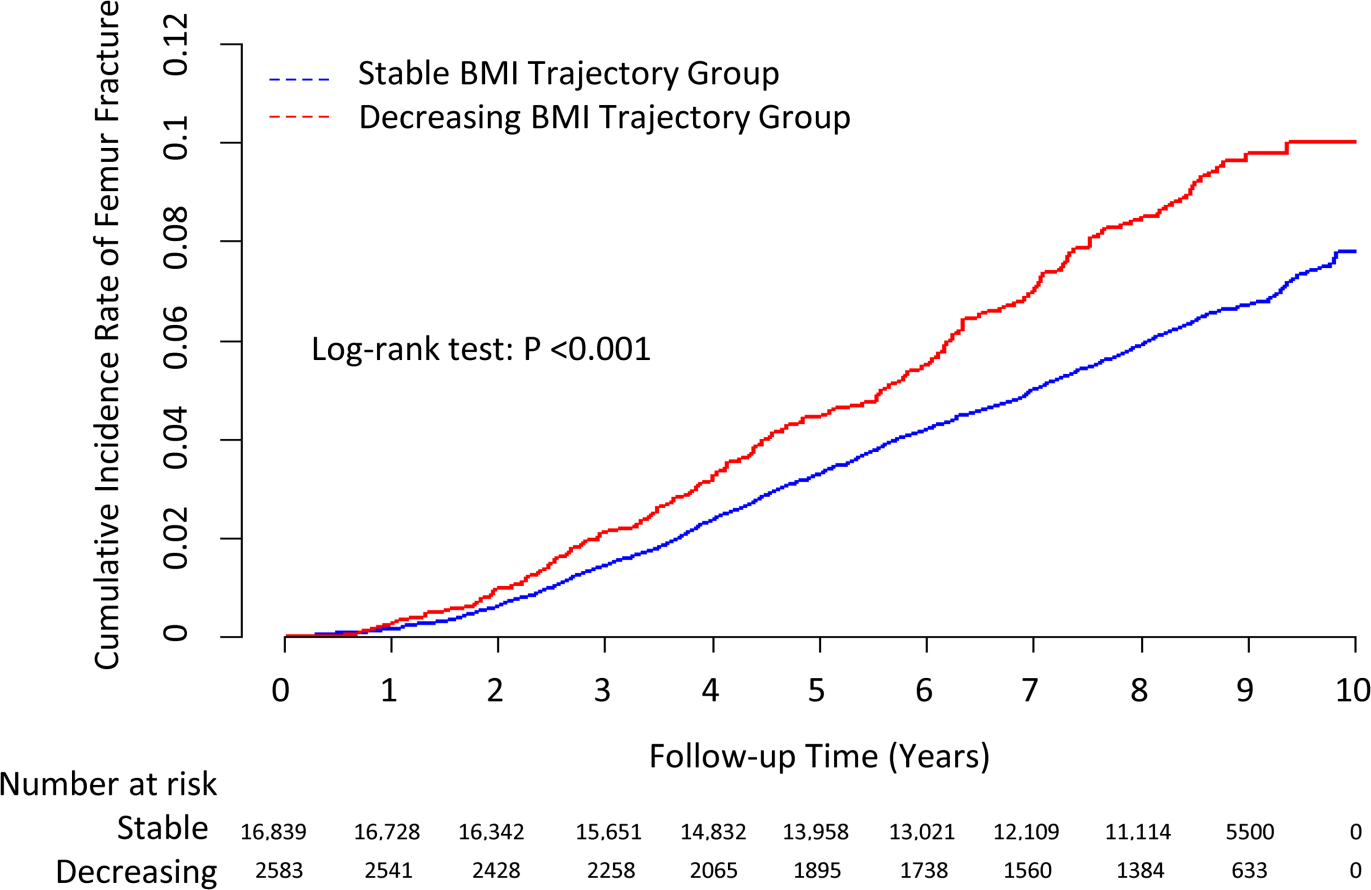
Cumulative incidence plots showing the rates of femur fracture incidence. BMI, body mass index.

During the median 8.46‐year event accrual period, 1156 participants (5.95%) experienced new‐onset femur fractures. The decreasing BMI group showed a significantly higher cumulative incidence of femur fractures than the stable group, as shown in the Kaplan–Meier curves (log‐rank *p* < 0.001; Figure 3).

Table 2 summarizes the associations between BMI change status and the risk of femur fractures. Compared with the individuals in the stable group, those in the decreasing BMI group had a higher incidence rate of femur fractures (10.50 vs. 7.58 per 1000 person‐years). In the unadjusted model, the decreasing group showed a significantly higher risk of femur fractures, with an HR of 1.41 (95% CI: 1.21–1.65, *p* < 0.001) compared to the stable group. This association remained statistically significant after multivariable adjustment. The adjusted HR (95% CI) was 1.20 (1.03–1.42, *p* = 0.024) in Model 1, 1.20 (1.02–1.41, *p* = 0.026) in Model 2 and 1.20 (1.02–1.41, *p* = 0.027) in Model 3.

**TABLE 2:** Risk of femur fracture based on the different BMI trajectory groups.

	BMI change			
	Stable	Decreasing		
Number, *n*	16 839	2583		
Incident femur fracture, *n*	969	187		
Follow‐up, person‐year	127773.7	17811.2		
IR	7.58	10.50		
Risk of femur fracture	HR	HR	95% CI	*p*
Unadjusted	1 (reference)	1.41	1.21–1.65	< 0.001
Model 1	1 (reference)	1.20	1.03–1.42	0.024
Model 2	1 (reference)	1.20	1.02–1.41	0.026
Model 3	1 (reference)	1.20	1.02–1.41	0.027

*Note:* Model 1: age, sex and BMI at baseline were adjusted. Model 2: age, sex, BMI at baseline, smoking, drinking and physical activity were adjusted. Model 3: age, sex, BMI at baseline, smoking, drinking, physical activity, DM, HTN, DLD and osteoporosis were adjusted.

Abbreviations: BMI, body mass index; CI, confidence interval; DLD, dyslipidaemia; HR, hazard ratio; HTN, hypertension; IR, incidence rate per 1000 person‐year; T2DM, type 2 diabetes mellitus.

Table S2 presents the adjusted HR (95% CI; *p*) for incident femur fracture for the relevant variables in Model 3. Per additional year of age, the HR was 1.08 (1.07–1.09; *p* < 0.001), and per unit increase in baseline BMI, it was 0.98 (0.96–1.00; *p* = 0.026); for categorical variables, the HR (95% CI; *p*) was 1.58 (1.35–1.86; *p* < 0.001) for women, 1.25 (0.95–1.64; *p* = 0.106) for current smokers, 0.91 (0.77–1.06; *p* = 0.215) for regular exercisers, 0.94 (0.77–1.14; *p* = 0.544) for alcohol drinkers, 1.12 (0.97–1.30; *p* = 0.117) for T2DM, 0.99 (0.88–1.16; *p* = 0.859) for HTN, 0.76 (0.64–0.90; *p* = 0.001) for DLD and 1.17 (1.02–1.33; *p* = 0.021) for osteoporosis compared with their respective counterparts.

### 3.3 Subgroup Analyses

**FIGURE 4:**
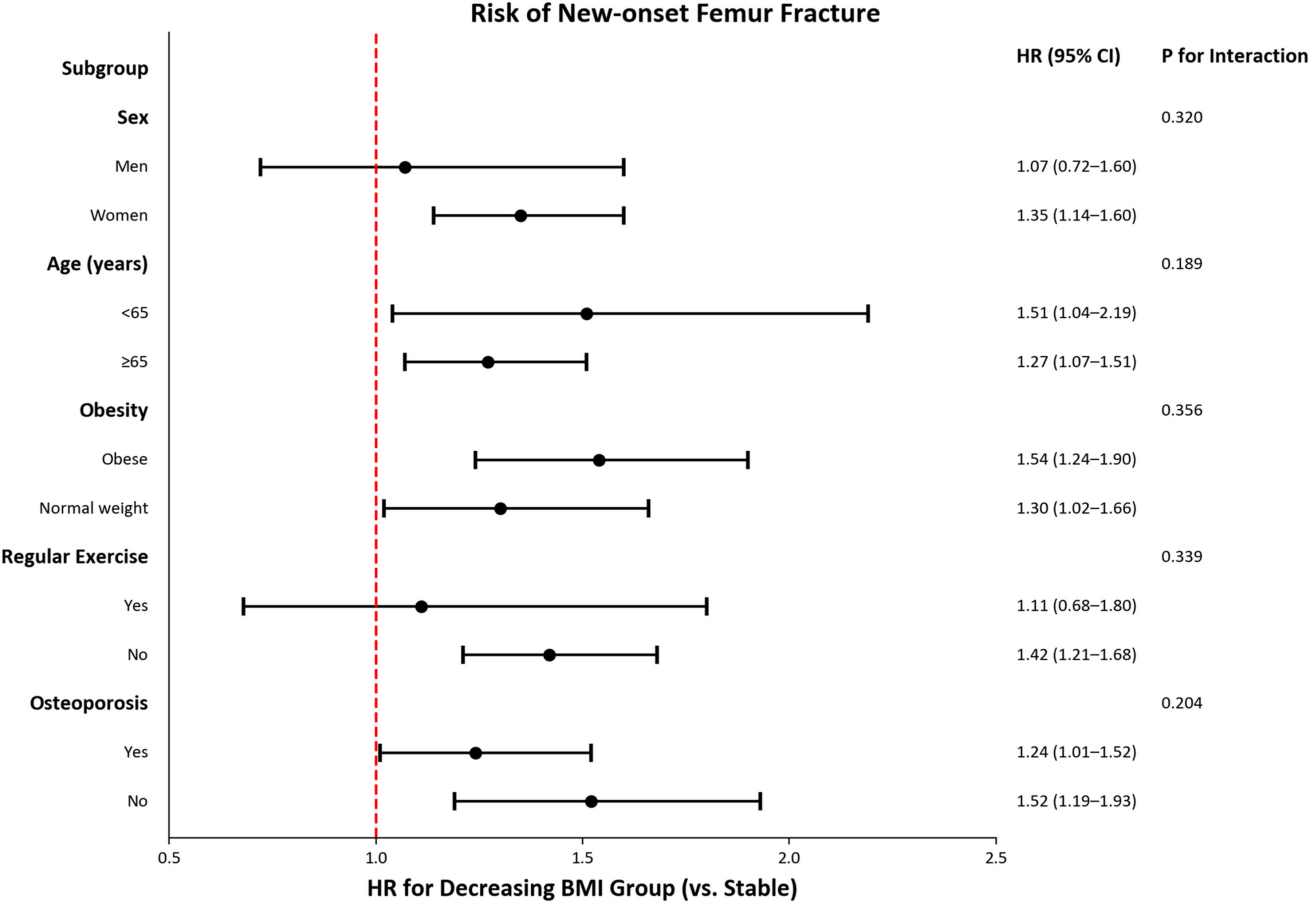
Subgroup analysis for the association between the different BMI trajectory groups (decreasing vs. stable) and the risk of new‐onset femur fracture among individuals with PD stratified by sex, age, obesity, regular exercise and osteoporosis. BMI, body mass index; CI, confidence interval; HR, hazard ratio; PD, Parkinson's disease.

Figure 4 presents a forest plot showing the association between the different BMI trajectory groups (decreasing vs. stable) and the risk of new‐onset femur fracture, stratified by sex, age, obesity, regular exercise and osteoporosis. The decreasing BMI group displayed a significantly elevated risk of femur fracture irrespective of age, obesity or osteoporosis. However, subgroup analyses by sex and physical activity revealed a different pattern. Women (HR 1.35; 95% CI 1.14–1.60; *p* = 0.001) and individuals without regular exercise (HR 1.42; 95% CI 1.21–1.68; *p* < 0.001) showed a notably higher fracture risk, whereas men (HR 1.07; 95% CI 0.72–1.60; *p* = 0.728) and regular exercisers (HR 1.11; 95% CI 0.68–1.80; *p* = 0.677) did not exhibit a significant difference between these trajectories.

## 4 Discussion

This study analysed the association between longitudinal changes in BMI and the risk of femur fracture in patients with PD using a large‐scale, nationwide cohort dataset. The results showed that individuals in the BMI decrease trajectory group had a significantly increased risk of femur fracture. Our findings align with and diverge from previous studies in two major areas: the relationship between baseline BMI and fracture risk and the influence of BMI changes over time.

Several studies have consistently shown an inverse association between baseline BMI and hip fracture risk in the general population [13, 14, 15]. In contrast, other studies have reported that a longitudinal reduction in BMI is associated with an increased risk of fractures [28, 29]. In this study, the decreasing BMI group exhibited a higher fracture risk despite having a higher baseline BMI. Even after adjusting for baseline BMI, this trend persisted. These findings suggest that among patients with PD, a temporal reduction in BMI may have a stronger influence on femur fracture risk than the absolute value of baseline BMI.

Although the precise mechanisms linking this progressive decline in BMI to fracture risk in PD remain incompletely understood, the association may be mediated by a combination of fall‐related vulnerability [2, 3] and alterations in bone metabolism [30]. Progressive weight loss is frequently observed in patients with PD and may indicate the possibility of sarcopenia [16]. While the direct link between longitudinal BMI decline and sarcopenia in PD remains to be fully delineated, sarcopenia has been consistently associated with disease severity [31], and BMI reduction typically parallels clinical progression [16]. Sarcopenia reduces lower‐limb strength and impairs balance, stability and proprioception, which collectively increase the risk of falling [32]. As the disease advances, disruptions in the basal ganglia and motor control systems further compromise locomotion, balance and coordination, potentially exacerbating the functional deficits associated with sarcopenia [1, 4, 16]. Therefore, among patients with PD, those exhibiting a decreasing BMI trajectory may be at particularly high risk for falls due to the combined effects of disease progression and the possibility of sarcopenia. Sarcopenia also impairs bone health, as muscle and bone are interconnected and influenced by common factors like physical activity, myokine‐related signalling pathways [33, 34]. Muscle contractions and mechanical load during physical activity provide essential stimuli for bone maintenance [33, 34]. When sarcopenia develops in the decreasing BMI group, this stimulation is diminished, which may accelerate bone loss and lead to osteoporotic changes. The associated loss of muscle mass also leads to reduced secretion of myokines, muscle‐derived bioactive substances involved in bone formation, remodelling and regeneration [35]. Moreover, patients with PD frequently present with nonmotor symptoms such as decreased appetite, dysphagia and constipation, which can lead to chronic nutritional deficiencies [1, 36]. In particular, inadequate intake of vitamin D and calcium is closely linked to decreased bone mineral density [36, 37]. Furthermore, long‐term use of dopaminergic medications, particularly levodopa, may increase serum homocysteine levels and reduce the absorption of vitamin B12 and folate, which may indirectly impair bone metabolism [38].

In the subgroup analysis of this study, a noteworthy finding was that the increased fracture risk associated with BMI reduction was not observed in men and in those who engaged in regular exercise. Because men typically have greater muscle mass and strength than women, they may be less susceptible to the negative effects of BMI reduction on femoral fracture risk [31, 39]. In addition, regular exercise helps preserve muscle mass, strength and balance [40], potentially minimizing the impact of BMI decline. Consequently, the maintenance of muscle function could have mitigated the increase in fracture risk in these subgroups. Collectively, these findings suggest that BMI reduction in patients with PD is not merely a numerical change in body weight but may serve as a clinical indicator reflecting osteosarcopenic pathophysiology. This condition likely contributes to increased fracture risk through both fall‐related vulnerability and impaired bone metabolism. Accordingly, longitudinal changes in BMI may be a practical marker for identifying patients at high risk of fracture.

This study has some limitations. First, detailed information on body composition, including muscle mass, fat distribution and bone mineral density, was not available in the dataset. Second, we could not assess the effects of certain medications, such as anti‐parkinsonian agents or glucocorticoids, which might influence the outcomes because of insufficient data. Third, the severity and progression of PD were not considered. Lastly, causal relationships between BMI changes and fracture risk cannot be definitively established owing to retrospective cohort study design. Despite these limitations, to the best of our knowledge, this is the first study to identify distinct longitudinal BMI trajectories and their associations with femur fracture risk among patients with PD using a nationwide population‐based cohort and data‐driven latent trajectory classification methods. These findings may inform future clinical risk stratification and preventive strategies.

In conclusion, a decreasing trajectory of BMI is associated with a higher risk of femur fracture in patients with PD. Serial BMI monitoring can provide a clinically meaningful measure for assessing fracture risk. Incorporating longitudinal BMI change into clinical practice may enable early intervention and personalized preventive strategies, ultimately improving long‐term outcomes in this population.

## Conflicts of Interest

The authors declare no conflicts of interest.

## Supporting information


**Table S1.** Estimation process for the most optimal number of BMI trajectory groups. Abbreviations: BMI, body mass index; BIC, Bayesian information criterion.
**Table S2.** Multivariable Cox proportional hazard regression model for the risk of femur fracture. Abbreviations: BMI, body mass index; HR, hazard ratio; CI, confidence interval; T2DM, type 2 diabetes mellitus; HTN, hypertension; DLD, dyslipidaemia. *Adjusted for age, sex, BMI at baseline, smoking, drinking, physical activity, DM, HTN, DLD and osteoporosis

## Data Availability

We do not have the authority to share the data because they were derived from public data provided by the Korean National Health Insurance Service.
