# Supplementary material for: Body Mass Index Changes and Femur Fracture Risk in Parkinson's Disease: National Cohort Study
Source: J Cachexia Sarcopenia Muscle. 2025 Jun 4;16(3):e13860. doi: 10.1002/jcsm.13860 (PMC12134786; doi:10.1002/jcsm.13860)
Supplement: Supplementary file 1 — Table S1. Estimation process for the most optimal number of BMI trajectory groups. Abbreviations: BMI, body mass index; BIC, Bayesian information criterion. Table S2. Multivariable Cox proportional hazard regression model for the risk of femur fracture. Abbreviations: BMI, body mass index; HR, hazard ratio; CI, confidence interval; T2DM, type 2 diabetes mellitus; HTN, hypertension; DLD, dyslipidaemia. *Adjusted for age, sex, BMI at baseline, smoking, drinking, physical activity, DM, HTN, DLD and osteoporosis [file JCSM-16-e13860-s001.docx]

**Supplementary Table 1.** Estimation process for the most optimal number of BMI trajectory groups

| Number of groups | Group 1 | Group 2 | Group 3 | Group 4 | Group 5 | BIC | Average probability of group membership |
| --- | --- | --- | --- | --- | --- | --- | --- |
| 2 | 16,839  (86.7%) | 2583  (13.3%) |  |  |  | -238,656.7 | 85.8 |
| 3 | 545  (2.8%) | 8257  (42.5%) | 10,620  (54.7%) |  |  | -238,103.5 | 74.5 |
| 4 | 641  (3.3%) | 12,202  (62.8%) | 2539  (13.1%) | 4040  (20.8%) |  | -238,082.9 | 62.1 |
| 5 | 76  (0.4%) | 6661  (34.3%) | 480  (2.5%) | 9612  (49.5%) | 2593  (13.4%) | -238,056.5 | 57.2 |

Abbreviations: BMI, body mass index; BIC, Bayesian information criterion.

Supplementary Table 2. Multivariable Cox proportional hazard regression model for the risk of femur fracture

|  | Risk of Femur Fracture | |
| --- | --- | --- |
|  | Adjusted^*^ | |
| Variables | HR (95% CI) | P |
| Decreasing BMI trajectory group (vs. stable) | 1.20 (1.02–1.41) | 0.027 |
| Women (vs. men) | 1.58 (1.35–1.86) | <0.001 |
| Age (per every year increment) | 1.08 (1.07–1.09) | <0.001 |
| Baseline BMI (per unit increment) | 0.98 (0.96–1.00) | 0.026 |
| Current smokers (vs. non-/ex-smokers) | 1.25 (0.95–1.64) | 0.106 |
| Regular exercisers (vs. non-regular exercisers) | 0.91 (0.77–1.06) | 0.215 |
| Alcohol drinkers (vs. non-drinker) | 0.94 (0.77–1.14) | 0.544 |
| DM (vs. non-DM) | 1.12 (0.97–1.30) | 0.117 |
| HTN (vs. non-HTN) | 0.99 (0.88–1.16) | 0.859 |
| DLD (vs. non-DLD) | 0.76 (0.64–0.90) | 0.001 |
| Osteoporosis (vs. non-osteoporosis) | 1.17 (1.02–1.33) | 0.021 |

Abbreviations: BMI, body mass index; HR, hazard ratio; CI, confidence interval; T2DM, type 2 diabetes mellitus; HTN, hypertension; DLD, dyslipidemia.

^*^Adjusted for age, sex, BMI at baseline, smoking, drinking, physical activity, DM, HTN, DLD, and osteoporosis
